# Moral Disengagement and Unethical Generative AI Use as the Chain Mediators Between Antagonistic Personality and Problematic Generative AI Use

**DOI:** 10.3390/bs16040500

**Published:** 2026-03-27

**Authors:** Kağan Kırcaburun, Pınar Özdemir

**Affiliations:** 1 Department of Educational Sciences, Faculty of Education, Düzce Üniversitesi, Düzce 81620, Türkiye; 2 Department of Psychological Counseling and Guidance, Faculty of Education, Düzce Üniversitesi, Düzce 81620, Türkiye

**Keywords:** artificial intelligence, moral disengagement, unethical artificial intelligence use, antagonistic traits, technology addiction

## Abstract

The rapid integration of generative artificial intelligence (GAI) tools into academic and professional contexts has raised concerns regarding unethical use and the potential development of problematic usage patterns. Drawing on personality and moral psychology frameworks, the present study examined the associations between antagonistic personality traits (narcissism, Machiavellianism, and psychopathy) and problematic (i.e., addictive) GAI use (PGAIU), as well as the chain mediating effect of moral disengagement and unethical GAI use (UGAIU). Data were collected from an adult sample (*N* = 491; 52% men; M_age_ = 43.92) using validated self-report measures. Path analysis indicated that narcissism exhibited significant direct and indirect associations with PGAIU. In contrast, Machiavellianism and psychopathy were indirectly related to PGAIU via moral disengagement and UGAIU but demonstrated non-significant total and direct effects. Multi-group analyses revealed broadly similar structural patterns across men and women, although some paths involving moral disengagement were significant only among men. A comparable pattern was also observed across age groups, with only minor variations in the mediation pathways. Overall, the findings highlight the central role of moral disengagement and unethical GAI-related behaviors in linking antagonistic personality traits to PGAIU.

## 1. Introduction

The rapid expansion of generative artificial intelligence (GAI) has fundamentally transformed how individuals engage with digital tasks. Rather than acting as passive consumers of online content, users now actively generate text, images, and conceptual ideas through simple natural language prompts (Kooli et al., 2025). GAI platforms (e.g., ChatGPT, Gemini) facilitate a more interactive and “human-like” engagement compared to traditional technologies (Yu et al., 2024). While providing significant utility, the highly responsive design of these tools may exert a strong psychological pull on users (Huang et al., 2025). Consequently, frequent GAI use may transcend functional purposes and evolve into a problematic or even addictive behavioral pattern for certain individuals (Kooli et al., 2025; Zhai et al., 2025).

As researchers have started to focus on this issue, various terms have been used in the literature, including problematic use of conversational AI (B. Hu et al., 2023), generative AI chatbots (Huang et al., 2025), AI chatbot dependency (Zhai et al., 2025; Zhang et al., 2024), problematic ChatGPT use (Yu et al., 2024), and addictive ChatGPT use (Deng & Deng, 2025). While often used interchangeably, these concepts possess distinct theoretical nuances. Addiction typically denotes severe, compulsive behavioral patterns accompanied by tolerance and withdrawal-like symptoms (Yankouskaya et al., 2025; Yu et al., 2024). Dependency highlights a deep functional or socio-emotional reliance where individuals feel incapable of functioning without the tool (Yankouskaya et al., 2025; Zhang et al., 2024). Conversely, problematic use serves as a broader umbrella term encompassing excessive and dysregulated engagement that impairs daily functioning, without necessarily meeting strict clinical diagnostic criteria for addiction (Yu et al., 2024). Furthermore, problematic GAI use differs fundamentally from general problematic technology use (e.g., internet or social media addiction). While traditional digital technologies often involve passive content consumption or act merely as communication channels between humans, generative AI functions as an active ‘cognitive partner’ (Yankouskaya et al., 2025). It provides highly personalized, context-aware interactions that not only foster cognitive miserliness by encouraging users to offload mental effort (Deng & Deng, 2025), but also simulate human-like empathy, leading to unique pseudosocial or parasocial bonds (B. Hu et al., 2023; Huang et al., 2025; Yankouskaya et al., 2025). To reduce conceptual confusion and capture this broader spectrum of dysregulated human-GAI interaction, the present study adopts the term problematic generative artificial intelligence use (PGAIU), which refers to difficulties in controlling GAI use and the experience of negative outcomes in daily life (Goh et al., 2025).

Existing research has linked PGAIU to heightened academic stress and performance expectations (Liu et al., 2026; Zhang et al., 2024), as well as diminished task performance and impaired critical thinking skills (Goh et al., 2025), together with strong cognitive reliance on GAI driven by information-seeking and efficiency motives (Zhai et al., 2025). Furthermore, PGAIU has been associated with increased emotional attachment to GAI (Zhai et al., 2025), greater loneliness, lower life satisfaction, and a stronger fear of missing out (FOMO; Goh et al., 2025), alongside elevated depression (Yu et al., 2024) and rumination (B. Hu et al., 2023). Despite these findings, the literature remains limited in two significant ways. First, empirical evidence has mostly relied on student samples from non-Western regions, leaving Western cohorts under-examined. Second, the role of antagonistic personality traits in the development of PGAIU is not yet well understood. While individuals with such traits may exhibit moral disengagement and unethical GAI use (UGAIU), this specific area has been largely neglected in current literature.

To address these gaps, the present study uses the Interaction of Person–Affect–Cognition–Execution (I-PACE) model as a theoretical framework (Brand et al., 2019). We argue that PGAIU is a complex behavior influenced by core personality characteristics. In this study, we examine whether narcissism, Machiavellianism, and psychopathy are related to higher PGAIU levels. We also test moral disengagement as a cognitive mechanism and UGAIU as a behavioral expression of unethical involvement. In doing so, this study provides a new contribution to the literature by clarifying how the antagonistic traits may shape individuals’ problematic interactions with GAI.

### 1.1. Antagonistic Traits and Problematic GAI Use

According to the I-PACE model, personality serves as a core predispositional factor in the emergence of specific technology-related and online problematic behaviors (Brand et al., 2019). Despite this theoretical significance, empirical investigations into the personality correlates of PGAIU remain remarkably scarce. To date, research has predominantly focused on the Big Five traits, with only one study identifying negative bivariate correlations between PGAIU and openness, agreeableness, conscientiousness, and extraversion (Goh et al., 2025). However, the potential exacerbating role of socially aversive traits—namely narcissism, Machiavellianism, and psychopathy—has not yet been empirically examined in the context of GAI. Considered as antagonistic traits (Simpson & Schermer, 2026), these personality characteristics capture maladaptive interpersonal tendencies and impairments in self-regulation that have been linked to an elevated risk of problematic technology use (Sindermann et al., 2018). Unlike traditional digital platforms, GAI offers unique affordances, such as instantaneous content generation and the capacity to project enhanced competence with minimal effort (Yu et al., 2024); these features may be particularly appealing to individuals with high antagonistic personality scores, potentially fostering compulsive patterns of engagement.

Specifically, narcissism involves an inflated self-view and a sense of entitlement (Muris et al., 2017). Narcissistic individuals may utilize GAI as a self-enhancement tool to gain admiration or outperform peers without corresponding effort, thereby increasing the risk of compulsive use. Machiavellianism reflects a strategic and instrumental approach to social interactions, characterized by manipulation and flexible moral reasoning (Jonason & Webster, 2010; Paulhus & Williams, 2002). From this perspective, GAI may be viewed as a powerful medium for strategic advantage or deceptive communication, reinforcing maladaptive engagement. Finally, psychopathy is marked by impulsivity and reduced self-control (Jonason & Webster, 2010), which may lead to difficulties in regulating the immediate and rewarding feedback provided by GAI systems.

Taken together, these trait-specific pathways point to a common prediction: antagonistic personality characteristics are likely to be positively associated with PGAIU. This expectation is further supported by a growing body of research linking antagonistic traits to problematic engagement across related technological contexts, including social media (narcissism; Kircaburun et al., 2018), online gaming (Machiavellianism; Sindermann et al., 2018), and generalized internet use (psychopathy; Lee & Lim, 2021). Although these associations have been documented in non-GAI contexts, the underlying mechanisms—self-enhancement, instrumental exploitation, and impulsive reward seeking—are not platform-specific and therefore are expected to extend to GAI technologies. The present study offers the first direct empirical examination of these relationships, addressing an important gap at the intersection of personality psychology and the emerging literature on PGAIU.

### 1.2. The Mediating Role of Moral Disengagement

According to social cognitive theory, moral disengagement can be conceptualized as a set of cognitive mechanisms through which individuals temporarily detach their internal moral standards from unethical or antisocial behaviors, thereby minimizing feelings of personal responsibility and preserving a positive self-image (Bandura, 2002). The tendency to rationalize or legitimize unethical behavior is a common feature of psychopathy and Machiavellianism, as both traits are fundamentally oriented toward self-interest, goal attainment, and personal gain, often pursued with little regard for the potential harm inflicted on others (Jonason & Webster, 2010; Paulhus & Williams, 2002). All three antagonistic traits have been linked to elevated moral disengagement, suggesting an increased likelihood of justifying unethical behavior (Erzi, 2020; Zhao et al., 2026).

Morally disengaged individuals are more likely to engage in academic misconduct within educational settings (Prince et al., 2025) as well as unethical behaviors (e.g., cheating, unethical decision-making) in the workplace (Newman et al., 2020). Once activated, moral disengagement creates the cognitive conditions necessary for unethical behavior to occur—and, we argue, for unethical GAI use specifically. In digital environments, GAI technologies introduce a form of psychological distance between the user and the ethical implications of their actions: the tool performs the work, the output appears legitimate, and accountability is diffused across human and algorithmic agents (Shaw, 2025; Sun et al., 2025). This architecture maps directly onto Bandura’s (2002) mechanisms of moral disengagement, particularly displacement of responsibility and distortion of consequences, rendering GAI a uniquely permissive context for morally disengaged individuals. Consequently, we expect moral disengagement to mediate the association between antagonistic traits and UGAIU.

Beyond its role as a cognitive enabler of unethical behavior, moral disengagement may also directly elevate the risk of PGAIU through a distinct pathway. Prior research has documented associations between moral disengagement and various forms of problematic technology use, including online gaming, social media, and generalized internet use (Colella et al., 2024; Kocabıyık, 2026; Xiao & Cheng, 2023). Importantly, much of this literature has examined moral disengagement as a consequence of problematic use—positing that excessive technology engagement gradually erodes ethical inhibitions. While this directionality is plausible, the predominantly cross-sectional and correlational nature of existing studies precludes definitive causal inference, and the reverse pathway remains equally theoretically defensible. Drawing on the I-PACE model (Brand et al., 2019), we position moral disengagement as a predispositional cognitive response that operates upstream of problematic use: individuals who habitually disengage their moral standards are less likely to exercise the self-regulatory restraint needed to moderate technology use, and more likely to exploit GAI’s mood-modifying and effort-reducing properties as a maladaptive coping strategy (Goh et al., 2025). This is consistent with evidence that morally disengaged individuals show impaired self-regulation and heightened negative affect (Coşkun, 2025; Lu et al., 2025), both of which are established risk factors for behavioral addiction (Brand et al., 2019). The present study therefore contributes to this literature by positioning moral disengagement as a theoretically grounded antecedent—rather than consequence—of PGAIU, and by embedding this directional claim within an integrative personality-based model.

### 1.3. The Mediating Role of Unethical GAI Use

Antagonistic personality traits have been consistently linked to a broad range of unethical behaviors across organizational, academic, and interpersonal contexts, including workplace misconduct, academic dishonesty, and deceptive practices (Baughman et al., 2014; O’Boyle et al., 2012; Williams et al., 2010). Critically, each trait appears to operate through a distinct motivational pathway: narcissism drives unethical conduct through inflated self-perceptions and a sense of entitlement that justifies norm violations, Machiavellianism through heightened sensitivity to deceptive opportunities and strategic self-interest, and psychopathy through emotional callousness and diminished responsiveness to ethical constraints (Harrison et al., 2018). Recent evidence has extended these associations to the GAI domain, with narcissism and psychopathy emerging as significant predictors of GAI-related academic misconduct among university students (Liang et al., 2025; Sun et al., 2025). However, these studies were confined to Chinese student samples and operationalized misconduct narrowly as academic dishonesty, leaving open the question of whether antagonistic traits predict a broader pattern of unethical GAI use—encompassing undisclosed reliance, misrepresentation of AI-generated outputs, and context-specific rationalization—among adult populations in Western contexts.

Unethical GAI use (UGAIU) refers to behaviors in which individuals employ generative AI in ways that bypass personal effort, misrepresent the origin of outputs, or otherwise violate standards of honesty and accountability expected in formal academic and professional contexts (Dolunay & Temel, 2024). We propose that UGAIU functions as a key mediating mechanism linking antagonistic traits to PGAIU and offer two complementary theoretical rationales for this pathway. First, drawing on the I-PACE model (Brand et al., 2019), specific use motivations and behavioral patterns that produce rewarding outcomes increase vulnerability to problematic technology use by inducing positive mood modification and reinforcing continued engagement. When antagonistic individuals use GAI unethically—successfully completing tasks with reduced effort, enhancing their apparent competence, or avoiding detection—the resulting goal attainment may serve as a powerful reinforcer that intensifies preoccupation with and psychological reliance on GAI (Zhai et al., 2025). In this sense, UGAIU does not merely co-occur with PGAIU but may actively generate the reward-driven cycle that sustains it. Second, repeated reliance on unethical use patterns may gradually undermine individuals’ capacity to complete formal tasks without GAI assistance, fostering a form of cognitive dependency that manifests through the salience, tolerance, and withdrawal symptoms characteristic of behavioral addiction (Brand et al., 2019; Kooli et al., 2025). Empirical support for this mechanism is evident in parallel research showing that antisocial and norm-deviating motivations for social media and gaming use—such as cyberbullying and cyberstalking—are associated with problematic engagement with those platforms (Kircaburun et al., 2018; Tang et al., 2020), suggesting that norm-violating forms of use may represent a broader transdiagnostic pathway to problematic technology engagement.

Furthermore, we propose that the pathway from antagonistic traits to PGAIU is serially mediated by moral disengagement and UGAIU. Moral disengagement provides the cognitive scaffolding—through mechanisms such as moral justification, diffusion of responsibility, and advantageous comparison—that enables antagonistic individuals to engage in UGAIU without experiencing the self-regulatory inhibition that ethical standards would otherwise impose (Bandura, 2002). UGAIU, in turn, initiates the reinforcement cycle described above, ultimately elevating the risk of PGAIU. This serial chain is theoretically grounded in social cognitive theory’s account of how distal personality dispositions translate into proximal behavioral outcomes through successive cognitive and motivational processes (Bandura, 2002), and is consistent with the I-PACE model’s hierarchical conceptualization of predispositional factors, affective-cognitive responses, and behavioral execution (Brand et al., 2019).

### 1.4. The Role of Gender

Extant literature consistently highlights gender-based differences in antagonistic personality traits, moral disengagement, and PGAIU. Men tend to exhibit higher levels of antagonistic traits compared to women (Craker & March, 2016; Muris et al., 2017). In parallel, men are more prone to moral disengagement, whereas women generally demonstrate greater sensitivity in differentiating ethical from unethical behaviors (Franke et al., 1997). Recent empirical studies further reveal that men report higher levels of PGAIU and are more likely to engage in problematic patterns of GAI use than women (Li et al., 2025; Yu et al., 2024). Taken together, these findings suggest that the proposed model may account for variance in PGAIU more effectively among men than women.

Based on the aforementioned rationale, we hypothesized that antagonistic traits would be both directly and indirectly related to PGAIU via moral disengagement and UGAIU, including a serial pathway from moral disengagement to UGAIU. Beyond testing these direct and indirect effects, the model was tested for the total sample as well as separately for men and women. In addition, exploratory analyses were conducted across age groups to examine whether the structural relationships differed by age. Finally, given that younger age and greater daily time spent on digital technologies have been consistently identified as key risk indicators for problematic technology use (Kircaburun et al., 2018), these variables were included as covariates into the model.

## 2. Methods

### 2.1. Participants and Procedure

We collected the data in two phases. In the first phase, a total of 360 participants were recruited for the exploratory factor analysis (EFA) conducted as part of the scale development process (49% men; M_age_ = 41.87, SD_age_ = 12.92; age range = 19–79 years). In the second phase (see Table 1), which involved confirmatory factor analysis (CFA) and subsequent statistical analyses, 491 participants took part in the study (52% men; M_age_ = 43.92, SD_age_ = 11.46; age range = 28–83 years). Consistent with widely cited guidelines for path analysis, sample sizes exceeding 200 are generally regarded as adequate for stable model estimation, indicating that the final subsample sizes in the present study were sufficient for the analyses conducted (Kline, 2016).

All participants were recruited via Prolific (prolific.com), a widely used and reliable online participant recruitment platform, and were provided with a link to an online survey. Prior to participation, all respondents were informed about the aims of the study and were assured that participation was voluntary and that all data would be treated confidentially and anonymously. All procedures involving human participants were conducted in accordance with the ethical standards of the institutional research committee and with the 1964 Helsinki Declaration and its later amendments. Informed consent was obtained electronically from all participants prior to their participation in the study.

### 2.2. Measures

First, participants were asked to provide demographic information, including gender, age, ethnicity, occupation, purpose of generative artificial intelligence (GAI) use, and frequency of daily GAI use. Subsequently, participants completed questionnaire measures related to problematic GAI use (PGAIU), antagonistic personality traits, moral disengagement, and unethical GAI use (UGAIU).

#### 2.2.1. Problematic Generative AI Use (PGAIUS)

The PGAIUS (B. Hu et al., 2023) was used to assess PGAIU. In the original version of the scale, the items refer to conversational AI (CAI); however, in the present study, references to CAI were replaced with GAI to capture participants’ problematic use of GAI technologies. The scale consists of six items rated on a five-point Likert scale (e.g., “*I felt an urge to use GAI more and more*.”). Item scores were averaged to create a single composite index of PGAIU. Evidence for the scale’s convergent and discriminant validity is reported in Table 2.

Internal consistency and reliability indices (Cronbach’s alpha, McDonald’s omega, average variance extracted [AVE], and composite reliability [CR]) are also provided in the same table.

In addition, a confirmatory factor analysis (CFA) was conducted in the present study to examine the factorial validity of all scales. The CFA results are presented in Table 3.

#### 2.2.2. Dark Triad Dirty Dozen Scale (DTDD)

Antagonistic personality traits were assessed using the DTDD (Jonason & Webster, 2010), which assesses three core constructs: narcissism (e.g., *“I tend to want others to admire me”*), Machiavellianism (e.g., *“I tend to manipulate others to get my way”*), and psychopathy (e.g., *“I tend to be cynical”*). Each personality dimension is assessed with four items rated on a nine-point Likert scale ranging from 1 (*strongly disagree*) to 9 (*strongly agree*). The results of the present analyses indicated that all three subscales demonstrated satisfactory reliability and validity in the current sample (see Table 2 and Table 3).

#### 2.2.3. Propensity to Morally Disengage Scale (PMDS)

Participants’ moral disengagement was assessed using the PMDS (Moore et al., 2012). The PMDS consists of eight items designed to capture individuals’ tendencies to cognitively justify or rationalize unethical behavior (e.g., *“People can’t be blamed for doing things that are technically wrong when all their friends are doing it too.”*). Responses are recorded on a seven-point Likert scale ranging from 1 (*strongly disagree*) to 7 (*strongly agree*). The results of the present analyses indicated that the scale demonstrated satisfactory internal consistency (see Table 2 and Table 3).

#### 2.2.4. Unethical Generative AI Use Scale (UGAIUS)

The UGAIUS was developed in the present study to assess individuals’ levels of UGAIU. Initially, an item pool of 23 items was generated based on an extensive review of the relevant literature and expert evaluations to ensure content validity. All items were rated on a five-point Likert scale ranging from 1 (*strongly disagree*) to 5 (*strongly agree*). EFA was conducted using principal axis factoring with varimax rotation with Kaiser normalization. Prior to factor extraction, sampling adequacy was confirmed. Items exhibiting cross-loadings above 0.20 across factors were systematically removed. As a result, seven items were eliminated, yielding a 16-item scale comprising four latent factors. (KMO = 0.88, *p* < 0.001, explaining 61.74% of the variance).

Subsequently, CFA was performed to further evaluate the factor structure. One factor was excluded due to weak inter-factor correlations and inadequate contribution to overall model fit, resulting in a more parsimonious and theoretically coherent solution. The final version of the scale consisted of 13 items loading on three factors, including Academic Integrity-Related AI Use (e.g., *“I tend to use GAI even when its appropriateness for a given task is uncertain.”*), Instrumental AI Use Orientation (e.g., *“I may use AI-generated text or content in formal tasks with only minimal modification.”*), and Moral Disengagement in AI Use (e.g., *“Using GAI is different from traditional forms of misconduct or misrepresentation.”*).

All items exhibited acceptable loadings on their respective factors in both analyses, with EFA factor loadings and CFA standardized factor loadings ranging from 0.54 to 0.83, supporting the factorial validity of the scale (see Table 3). As shown in Table 2, the UGAIUS and its subdimensions demonstrated satisfactory composite reliability (CR = 0.75–0.94). Average variance extracted (AVE) values ranged from 0.40 to 0.60, exceeding the minimum acceptable threshold, thereby supporting the convergent validity of the scale and its subfactors (Lam, 2012).

### 2.3. Data Analytic Strategy

The scale development process for the UGAIUS involved both EFA and CFA. EFA was performed using principal axis factoring with varimax rotation. Sampling adequacy was assessed prior to factor extraction. CFA was subsequently conducted to examine the factorial validity of all multi-item measures included in the study, including the DTDD, UGAIUS, PGAIUS, and the PMDS. Model fit was evaluated using multiple fit indices, including the comparative fit index (CFI), goodness of fit index (GFI), root mean square error of approximation (RMSEA), and standardized root mean square residual (SRMR). Additionally, group comparison based on gender was performed using independent samples *t*-tests. Common method bias was also tested via Harman’s single-factor test by examining whether a single factor accounted for the majority of the variance.

To explore potential age-related differences, participants were categorized into two age groups (28–42 years and 43–83 years). Independent samples *t*-tests were conducted to examine group differences in the study variables. In addition, the proposed mediation model was tested separately for both age groups to explore potential differences in the pattern of relationships.

**Figure 1:**
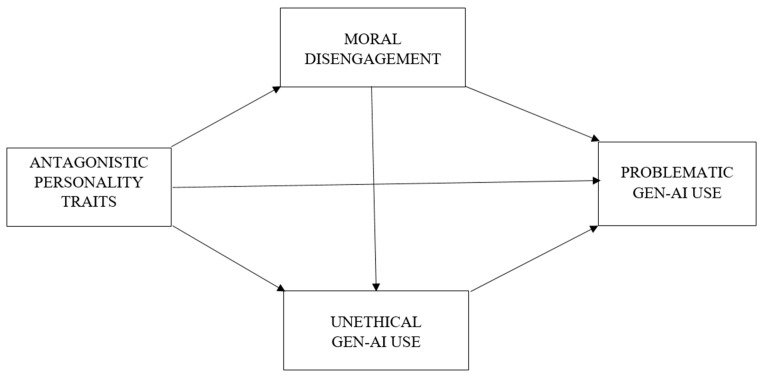
Theoretical model. Note. GEN-AI = generative artificial intelligence.

To test the hypothesized direct and indirect relationships among antagonistic personality traits, moral disengagement, UGAIU, and PGAIU (see Figure 1), path analysis was employed using the second-phase sample (N = 491). Indirect effects were examined using bias-corrected bootstrapping procedures with 10,000 resamples, and 95% confidence intervals were reported. Daily GAI use frequency, age, and gender were included as control variables in the model. In addition, multi-group analyses were conducted to examine potential gender differences in the pathways. Data analyses were conducted using SPSS 26 and AMOS 24.

## 3. Results

### 3.1. Descriptive Statistics

Table 2 presents the means, standard deviations, reliability coefficients, and Pearson correlation coefficients among the study variables. All scales demonstrated acceptable to excellent internal consistency (Cronbach’s α and McDonald’s ω values ≥ 0.75). All skewness and kurtosis values were within acceptable limits for univariate normality (Kline, 2016). Considering the satisfactory CR values, AVE values exceeding 0.40 were deemed acceptable, in line with previous methodological recommendations (Lam, 2012).

Problematic generative artificial intelligence (GAI) use was positively and moderately correlated with unethical GAI use (*r* = 0.42, *p* < 0.001). At the subscale level, Problematic GAI use (PGAIU) showed significant positive associations with academic-related unethical GAI use (*r* = 0.41, *p* < 0.001), instrumental AI use orientation (*r* = 0.26, *p* < 0.001), and moral disengagement in AI use (*r* = 0.37, *p* < 0.001). Moral disengagement was positively associated with all unethical GAI use (UGAIU) dimensions (*r*s ranging from 0.25 to 0.41, *p* < 0.001). Regarding personality traits, narcissism and Machiavellianism were positively correlated with PGAIU (*r*s = 0.33 and 0.14, respectively). Similarly, all antagonistic traits were significantly related to UGAIU and moral disengagement, with Machiavellianism showing particularly robust correlations with moral disengagement (*r* = 0.53, *p* < 0.001) and UGAIU dimensions.

Furthermore, independent-samples *t*-tests were conducted to examine gender differences (women = 1, men = 2) across the study variables (results not shown in tabular form). The results indicated that men scored significantly higher than women on Machiavellianism (*t*[489] = −2.49, *p* < 0.05), psychopathy (*t*[489] = −2.93, *p* < 0.01), narcissism (*t*[489] = −2.10, *p* < 0.05), UGAIU (*t*[489] = −3.03, *p* < 0.01), and moral disengagement (*t*[489] = −2.83, *p* < 0.01). Although men also reported higher levels of PGAIU, this difference did not reach statistical significance (*t*[489] = −1.82, *p* = 0.07). To further explore potential age-related differences, the proposed mediation model was tested separately for two age groups (28–42 years and 43–83 years). Independent samples *t*-tests showed that the younger group reported significantly higher PGAIU scores than the older group (*t*[489] = 4.84, *p* < 0.01), while no significant differences were observed for the other variables.

### 3.2. Common Method Bias

In line with the guidelines of Podsakoff et al. (2003), Harman’s one-factor test was conducted as an initial assessment of common method bias. All study variables were entered into an exploratory factor analysis using principal component analysis with varimax rotation, constrained to a single-factor solution. The results showed that the single factor accounted for 27.81% of the total variance. While relying solely on this procedure has recognized limitations (Podsakoff et al., 2003), recent contemporary literature demonstrates that Harman’s test remains a valid and effective indicator when a study is built upon strong theorizing (Kock, 2021; Kock & Dow, 2025). Therefore, given our well-developed theoretical model, this result provides preliminary support that a single factor does not account for the majority of the variance. A more detailed discussion on the limitations of this approach and recommendations for future research are provided in the Limitations section.

### 3.3. Model Testing

**Figure 2:**
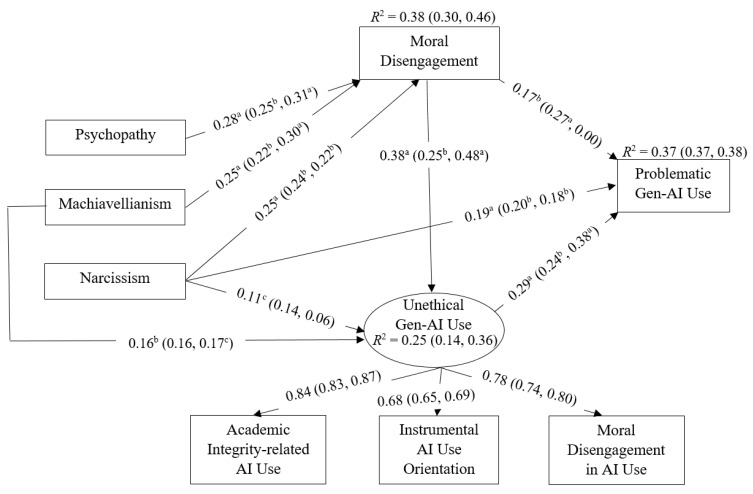
Final model of the significant path coefficients. Note. Latent constructs in the model are represented by circles, while observed indicators are illustrated as rectangles. Daily Gen-AI (generative artificial intelligence) use (*β* = 0.29, *p* < 0.001; 95% CI [0.20, 0.38]), gender (women = 1, men = 2; *β* = −0.02, *p* > 0.05; 95% CI [−0.09, 0.06]), and age (*β* = −0.12, *p* < 0.01; 95% CI [−0.19, −0.05]) were included in the model as covariates. Standardized path estimates are presented for the total sample outside the brackets; estimates for men and women are reported on the left and right sides of the brackets, respectively. For presentation purposes, several paths were omitted from the figure. Specifically, non-significant associations and covariances among the variables are not displayed. ^a^ *p* < 0.001, ^b^ *p* < 0.01, ^c^ *p* < 0.05.

Table 3 demonstrates the fit indices of the models tested in the path analyses, all of which indicated acceptable to good model fit according to commonly recommended criteria. Table 4 presents the standardized total, direct, and indirect effects, and Figure 2 illustrates the final model. Narcissism showed a significant positive total (*β* = 0.29, *p* < 0.001; 95% CI [0.20, 0.39]) and direct association (*β* = 0.19, *p* < 0.001; 95% CI [0.10, 0.28]) on PGAIU in the overall sample and across gender groups. Moral disengagement also exhibited a significant total association on PGAIU (*β* = 0.28, *p* < 0.001; 95% CI [0.16, 0.39]), which was stronger among men than women, whereas its direct relationship was non-significant among women. Machiavellianism and psychopathy did not show significant direct associations with PGAIU. However, both traits exerted significant indirect links via moral disengagement and UGAIU (Machiavellianism: *β* = 0.12, *p* < 0.001; psychopathy: *β* = 0.05, *p* < 0.05), indicating full mediation. Nevertheless, their total relationships with PGAIU were non-significant.

When the mediation model was examined separately for each age group (not depicted in the figure), the overall pattern of relationships remained largely consistent across groups. The only notable difference concerned the association between moral disengagement and PGAIU: in the younger group, this relationship became fully indirect through the mediators (*β* = 0.14, *p* < 0.001; 95% CI [0.06, 0.24]), whereas in the older group the partial mediation pattern observed in the total sample remained (*β* = 0.09, *p* < 0.01; 95% CI [0.03, 0.17]). In addition, age-specific differences emerged in the pathway linking Machiavellianism to UGAIU. In the younger group, this association was partially mediated by moral disengagement (*β* = 0.11, *p* < 0.01; 95% CI [0.05, 0.18]), whereas in the older group the relationship between Machiavellianism and UGAIU was fully mediated by moral disengagement (*β* = 0.09, *p* < 0.01; 95% CI [0.03, 0.17]). As shown in Figure 2, the overall model accounted for a substantial proportion of variance in PGAIU (*R*^2^ = 0.37). In addition, moral disengagement *(R*^2^ = 0.38) and UGAIU (*R*^2^ = 0.25) were significantly explained by personality traits included in the model. Daily GAI use was positively associated with PGAIU (*β* = 0.29, *p* < 0.001; 95% CI [0.20, 0.37]), whereas age showed a small but significant negative association (*β* = −0.12, *p* < 0.01; 95% CI [−0.18, −0.06]). Controlling for gender did not meaningfully alter the pattern of structural relationships in the total sample.

## 4. Discussion

Problematic generative artificial intelligence use (PGAIU) has recently emerged as a maladaptive form of technology engagement attracting growing scholarly attention. The present study provides novel contributions to this area via identifying key direct and indirect pathways in which predispositional factors (antagonistic personality traits) and cognitive mechanisms (moral disengagement and unethical generative artificial intelligence use; UGAIU) are implicated in elevated PGAIU among men and women. Specifically, narcissism was both directly and indirectly associated with PGAIU via moral disengagement and UGAIU. Machiavellianism and psychopathy were indirectly related to PGAIU via these cognitive processes, although their overall associations were not significant. Moral disengagement was directly and indirectly related to PGAIU in the total sample and among men, whereas it showed only an indirect association via UGAIU among women.

As expected, narcissism was positively associated with PGAIU in the total sample as well as among men and women. To our knowledge, this is the first empirical study to demonstrate a link between narcissism and PGAIU. This finding aligns with existing evidence showing that narcissism is related to problematic engagement with other technological platforms (Kircaburun et al., 2018). Prior work has indicated that individuals high in narcissism tend to use social media and gaming for self-enhancement and impression management (e.g., maintaining a favorable self-image) and as a means of escaping real-world demands (McCain & Campbell, 2018; Tang et al., 2020). In the context of GAI use, narcissistic individuals may be more vulnerable to PGAIU when attempting to outperform others or gain admiration in academic or professional tasks, thereby fulfilling personality-driven psychological needs for superiority and self-validation (Muris et al., 2017). However, not all narcissistic engagement with GAI reflects unethical use or moral disengagement; narcissism can also be related to productivity, achievement, and self-enhancement goals that are normatively acceptable (Jonason & Webster, 2010).

The association between narcissism and PGAIU was partially accounted for by higher moral disengagement, UGAIU, and moral disengagement-related UGAIU in the total sample and among men. However, for women, the relationship between narcissism and PGAIU emerged only indirectly via moral disengagement-related UGAIU, demonstrating that only a serial mediation pathway functioned in this subgroup. The direct association between narcissism and moral disengagement aligns with some previous studies reporting a similar link (Jones et al., 2017), yet contrasts with others suggesting that Machiavellianism and psychopathy emerge as stronger predictors of moral disengagement when examined concurrently (Egan et al., 2015). It may be that individuals high in narcissistic traits in the present study tended to justify their own actions to protect their self-image, rationalize entitlement-driven behavior, and downplay ethical boundaries when pursuing personal goals (Jones et al., 2017).

Contrary to expectations, Machiavellianism was only indirectly related to PGAIU despite showing significant positive correlations at the bivariate level. This likely reflects the shared variance among antagonistic traits, with narcissism capturing the central motivational drivers of engagement. It appears that when narcissistic motives such as self-enhancement and achievement orientation are accounted for, the additional contribution of manipulative (Machiavellian) and impulsive–callous (psychopathic) tendencies diminishes. Thus, narcissism may be the most proximal antagonistic trait linked to problematic use in the context of GAI use. This pattern also aligns with the I-PACE framework (Brand et al., 2019), which posits that each form of technology-related problematic behavior is shaped by a distinct constellation of individual characteristics, helping to clarify both the shared and unique mechanisms underlying different problematic engagement pathways.

Moral disengagement fully mediated the association between psychopathy and UGAIU in both men and women and partially mediated the link between Machiavellianism and UGAIU among women. Moral disengagement fully accounting for the associations between Machiavellianism and psychopathy with UGAIU is consistent with prior research showing that moral disengagement mediates the influence of these traits on unethical decision-making and behavior across other domains (Egan et al., 2015). Additional mechanisms (e.g., context-driven strategic motives, instrumental utility, or perceived performance benefits) may help explain why higher Machiavellian tendencies associate with greater UGAIU among women. Women’s Machiavellianism scores correlate with anxious personality features (Czibor et al., 2017), suggesting that emotional vulnerability rather than purely strategic manipulation may also contribute to UGAIU. Interestingly, age-specific differences also emerged in the Machiavellianism–UGAIU pathway, such that the association was fully mediated by moral disengagement among older participants but only partially mediated among younger users. This pattern may suggest that unethical GAI use among older individuals is more strongly dependent on cognitive moral justification processes, whereas younger users may engage in such behaviors through additional motivations (e.g., experimentation, instrumental convenience, or normative flexibility in digital environments).

Partially consistent with expectations, the association between moral disengagement and PGAIU was partly accounted for by UGAIU in the total sample and among men and fully accounted for by UGAIU among women. Although this is the first study to document a direct link between moral disengagement and UGAIU in the general population, the pattern aligns with evidence showing that morally disengaged individuals are more prone to misconduct in educational contexts and unethical behaviors in professional environments (Newman et al., 2020; Prince et al., 2025). Given that digital environments offer fewer immediate social sanctions and lower risk of detection than face-to-face settings (Ogunfowora et al., 2022), morally disengaged individuals may view the use of GAI for inappropriate purposes as more permissible and experience reduced guilt or responsibility (Theoharakis et al., 2025). Future research should investigate which additional mechanisms explain the moral disengagement–PGAIU association among men where UGAIU accounted for this link only partially. We can only speculate that morally disengaged men may have shown higher PGAIU not only for goal-oriented purposes, but also as a way to cope with negative feelings (Coşkun, 2025; Goh et al., 2025).

Additional exploratory analyses considering age groups revealed broadly similar patterns across age categories. Although younger participants reported higher levels of PGAIU, the overall mediation structure remained largely consistent. However, the relationship between moral disengagement and PGAIU was fully indirect in the younger group, whereas a partial mediation pattern was observed among older participants, suggesting that age may slightly shape the pathways linking moral disengagement to PGAIU. One possible explanation is that younger individuals may be more likely to translate morally disengaged cognitions into problematic GAI engagement primarily through specific unethical use practices (Song & Liu, 2025). In other words, for younger users, moral disengagement may first facilitate the justification of unethical GAI use behaviors, which subsequently increases the likelihood of problematic engagement with generative AI tools (Karakuş et al., 2025). In contrast, older individuals may engage in PGAIU through additional pathways beyond unethical use practices, resulting in a partially mediated relationship.

Although the Unethical Generative AI Use Scale (UGAIUS) and the Propensity to Morally Disengage Scale are theoretically related, they capture conceptually distinct constructs. Moral disengagement reflects generalized cognitive mechanisms that enable individuals to justify unethical behavior across a wide range of contexts, whereas UGAIUS focuses specifically on behavioral tendencies related to the unethical use of GAI systems. Nevertheless, because the scale validation and hypothesis testing were conducted within the same dataset, some degree of shared method variance or conceptual overlap cannot be entirely ruled out. Future research would benefit from further validating the UGAIUS using independent samples and additional methodological approaches to strengthen evidence for its discriminant validity.

In line with expectations, UGAIU was positively associated with PGAIU across all groups. This finding provides the first direct empirical evidence for a pathway that has thus far been theoretically assumed but not tested: that norm-violating patterns of GAI use constitute a behavioral antecedent of problematic engagement, rather than merely a co-occurring symptom. The result is consistent with parallel evidence from social media research, where antisocial use motivations—including cyberbullying and cyberstalking—have been shown to predict problematic social media use (Kircaburun et al., 2018), suggesting that norm-deviating use may function as a transdiagnostic pathway to problematic technology engagement across platforms. Theoretically, this finding is well accommodated by the I-PACE model’s account of how specific use patterns generate positive mood modification and goal attainment, thereby reinforcing continued and escalating engagement (Brand et al., 2019). When unethical GAI use successfully serves individuals’ academic or professional goals—reducing effort, enhancing apparent competence, or evading detection—the resulting reinforcement is likely to increase preoccupation with GAI and erode individuals’ capacity to complete formal tasks without AI assistance, progressively consolidating the salience, tolerance, and withdrawal-like features characteristic of PGAIU.

The consistency of this association across men and women suggests that the UGAIU–PGAIU pathway operates independently of gender, implying that interventions targeting unethical use patterns may be broadly effective regardless of the population subgroup. Future research should examine the temporal dynamics of this relationship using longitudinal designs, and investigate whether specific dimensions of UGAIU—such as undisclosed reliance versus active misrepresentation—differentially predict the onset and severity of PGAIU. Importantly, the moderate association between moral disengagement and UGAIUS observed in the present study supports the conceptual distinction between a general propensity to morally disengage and context-specific unethical AI use behaviors. In addition, the theoretically consistent associations observed between UGAIUS and related constructs provide preliminary support for the construct validity of the scale.

### Limitations

A number of limitations should be considered when evaluating the present results. To begin with, participants were English-speaking adults predominantly residing in Western countries, which limits the extent to which these findings can be transferred to other cultural or linguistic groups. Additional studies conducted with more diverse samples will be necessary to assess whether the same patterns emerge elsewhere. Moreover, a notable limitation concerns the directionality of the moral disengagement–PGAIU relationship. Although the present model positions moral disengagement as a theoretically grounded antecedent of PGAIU, consistent with the I-PACE framework, the cross-sectional design does not permit causal inference. The reverse pathway—whereby problematic GAI use gradually erodes moral standards—is equally plausible and cannot be ruled out on the basis of the present data. Future longitudinal and experience-sampling studies tracking moral disengagement and PGAIU across multiple time points would be essential to empirically adjudicate this directionality. Lastly, all variables were assessed using self-reported information provided by volunteers, which raises the possibility of biased responding or non-representative sampling. Future investigations should seek to include multiple forms of evidence—such as behavioral indicators, interview data, or mixed-method approaches—and recruit larger, more heterogeneous populations. Another limitation concerns the potential influence of common method variance, as all variables were measured using self-report instruments within a single survey. Although Harman’s single-factor test suggested that a single factor did not account for the majority of variance, this procedure has recognized limitations and should not be considered a definitive test of common method bias. Future studies could employ additional strategies, such as marker variables or confirmatory factor analytic approaches that model a latent method factor, to more rigorously assess potential method effects.

## 5. Conclusions

Despite these limitations, the present study is the first to simultaneously investigate antagonistic personality traits, moral disengagement, and UGAIU in relation to the development of PGAIU tendencies. Our results point to potentially abusive, antisocial, and maladaptive patterns of GAI use by identifying notable direct and indirect relations among these constructs. Given ongoing discussions surrounding UGAIU- and GAI-related misconduct (Shaw, 2025), these associations merit particular attention. Importantly, the pattern of results highlights the value of treating PGAIU as a distinct construct rather than merely an extension of general technology overuse. Accordingly, the study highlights the need to translate this understanding into preventive strategies and responsible use guidelines to mitigate harmful patterns of GAI engagement across academic and professional contexts.

## Figures and Tables

**Table 1:** Participants’ demographic characteristics and generative AI use behavior.

Variable		N	%
Gender			
	Men	257	52.3
	Women	234	47.7
Ethnicity			
	Caucasian	336	68.4
	Black/African	84	17.1
	Asian	36	7.3
	Hispanic/Latino	16	3.3
	Mixed	13	2.6
Occupation			
	Full-time	299	60.9
	Part-time	64	13.0
	Unemployed	62	12.6
	Self-employed	58	11.8
	Student	8	1.6
AI use purpose			
	General knowledge	246	50.1
	Work/education	206	42.0
	Social interaction	20	4.1
	Escape from real life	19	3.9
Daily GAI use			
	Rarely use GAI	3	0.6
	Less than 1 h	204	41.5
	Between 1–2 h	170	34.6
	Between 2–3 h	62	12.6
	Between 3–4 h	22	4.5
	Between 4–5 h	15	3.1
	More than 5 h	15	3.1

**Table 2:** Mean scores, standard deviations, and Pearson’s correlations of the study variables (N = 491).

	ω	CR	AVE	α	1	2	3	4	5	6	7	8	9
Problematic Gen-AI use	0.85	0.85	0.48	0.84	-								
2. UGAIU_Total	0.92	0.94	0.54	0.92	0.42 ***	-							
3. UGAIU_AIRAIU	0.91	0.91	0.60	0.91	0.41 ***	0.94 ***	-						
4. UGAIU_IAIUO	0.79	0.79	0.56	0.79	0.26 ***	0.76 ***	0.58 ***	-					
5. UGAIU_MDAIU	0.75	0.75	0.43	0.75	0.37 ***	0.83 ***	0.65 ***	0.56 ***	-				
6. Moral disengagement	0.84	0.84	0.40	0.84	0.31 ***	0.42 ***	0.39 ***	0.25 ***	0.41 ***	-			
7. Narcissism	0.86	0.85	0.60	0.86	0.33 ***	0.30 ***	0.30 ***	0.17 ***	0.25 ***	0.43 ***	-		
8. Machiavellianism	0.86	0.87	0.63	0.86	0.14 **	0.33 ***	0.32 ***	0.21 ***	0.27 ***	0.53 ***	0.43 ***	-	
9. Psychopathy	0.83	0.85	0.60	0.82	0.06	0.22 ***	0.22 ***	0.13 **	0.18 ***	0.51 ***	0.28 ***	0.63 ***	-
10. Gender					0.08	0.14 **	0.11 *	0.11 *	0.14 **	0.13 **	0.10 *	0.11 *	0.13 **
11. Age					−0.18 ***	−0.10 *	−0.13 **	0.05	−0.11 *	−0.04	−0.06	−0.04	−0.08
12. Daily time spent					0.39 ***	0.23 ***	0.22 ***	0.20 ***	0.14 **	0.04	0.09	0.00	−0.07
*Mean score*					1.84	38.60	16.81	3.51	2.81	2.15	3.54	3.29	2.69
*Standard deviation*					0.72	11.52	6.88	0.92	0.90	0.94	1.81	1.80	1.61
*Skewness*					1.01	−0.03	0.28	−0.83	−0.20	0.81	0.38	0.44	1.01
*Kurtosis*					0.78	−0.57	−0.86	0.38	−0.45	0.35	−0.54	−0.78	0.46

Note. ω = McDonald’s Omega reliability coefficient; CR = composite reliability; AVE = average variance extracted; α = Cronbach’s alpha reliability coefficient; Gen-AI = generative artificial intelligence; UGAIU = unethical Gen-AI use; AIRAIU = academic integrity-related AI use; IAIUO = instrumental AI use orientation; MDAIU = moral disengagement in AI use. * *p* < 0.05, ** *p* < 0.01, *** *p* < 0.001.

**Table 3:** Model fit indices for CFA and path analyses across the total sample and gender groups, with recommended cut-off values.

Fit Indices	Reference Values	Confirmatory Factor Analyses (CFA)				Path Analyses		
		PGAIUS (N = 491)	MDS (N = 491)	DTDD (N = 491)	UGAIUS (N = 491)	Total Sample (N = 491)	Men (N = 257)	Women (N = 234)
χ^2^/df	≤5 (Byrne, 2010)	3.60	3.77	4.09	3.00	2.82	2.99	1.59
RMSEA	≤0.08 (L. T. Hu & Bentler, 1999)	0.07	0.07	0.07	0.07	0.06	0.09	0.05
SRMR	≤0.05 (Byrne, 2010)	0.03	0.04	0.05	0.04	0.02	0.04	0.03
CFI	≥0.90 (L. T. Hu & Bentler, 1999)	0.98	0.96	0.96	0.96	0.98	0.96	0.99
GFI	≥0.90 (L. T. Hu & Bentler, 1999)	0.98	0.96	0.94	0.94	0.98	0.97	0.98

Note. PGAIUS = Problematic Generative Artificial Intelligence Use Scale; MDS = Moral Disengagement Scale; DTDD = Dark Triad Dirty Dozen; UGAIUS = Unethical Generative Artificial Intelligence Use Scale.

**Table 4:** Standardized estimates of total, direct, and indirect effects on problematic GAI use for overall sample and men and women.

	Effect (S.E.)		
	All Sample	Men	Women
Narcissism → PGAIU (total effect)	0.29 *** (0.05)	0.31 *** (0.07)	0.24 ** (0.07)
→ PGAIU (direct effect)	0.19 *** (0.05)	0.20 ** (0.07)	0.18 * (0.07)
→ PGAIU (indirect effect)	0.10 *** (0.02)	0.11 *** (0.03)	0.06 * (0.03)
→ MD → PGAIU	0.02 ** (0.01)	0.03 *** (0.01)	0.01 (0.02)
→ UGAIU → PGAIU	0.01 * (0.01)	0.01 * (0.01)	0.01 (0.01)
→ MD → UGAIU → PGAIU	0.01 * (0.01)	0.01 ** (0.00)	0.02 *** (0.02)
Machiavellianism → PGAIU (total effect)	0.03 (0.06)	0.01 (0.07)	0.06 (0.08)
→ PGAIU (direct effect)	−0.09 (0.06)	−0.10 (0.07)	−0.06 (0.08)
→ PGAIU (indirect effect)	0.12 *** (0.03)	0.11 ** (0.04)	0.11 ** (0.04)
→ MD → PGAIU	0.02 ** (0.01)	0.02 ** (0.01)	0.00 (0.01)
→ UGAIU → PGAIU	0.02 ** (0.01)	0.02 (0.01)	0.03 * (0.02)
→ MD → UGAIU → PGAIU	0.01 ** (0.00)	0.01 ** (0.00)	0.02 *** (0.01)
Psychopathy → PGAIU (total effect)	−0.03 (0.06)	−0.08 (0.08)	0.03 (0.08)
→ PGAIU (direct effect)	−0.08 (0.06)	−0.14 (0.07)	−0.00 (0.08)
→ PGAIU (indirect effect)	0.05 (0.03)	0.06 *** (0.04)	0.03 ** (0.05)
→ MD → PGAIU	0.01 (0.01)	0.07 *** (0.01)	0.01 (0.01)
→ MD → UGAIU → PGAIU	0.01 (0.01)	0.02 ** (0.00)	0.06 *** (0.02)
MD → PGAIU (total effect)	0.28 *** (0.06)	0.33 *** (0.07)	0.18 * (0.09)
→ PGAIU (direct effect)	0.17 ** (0.05)	0.27 *** (0.07)	0.00 (0.10)
→ UGAIU → PGAIU (indirect effect)	0.11 *** (0.03)	0.06 ** (0.03)	0.18 *** (0.05)

Note. GAI = generative artificial intelligence; PGAIU = Problematic Gen-AI Use; UGAIU = Unethical Gen-AI Use; MD = Moral Disengagement. * *p* < 0.05, ** *p* < 0.01, *** *p* < 0.001.

## Data Availability

Data supporting reported results can be found at EFA dataset and CFA dataset.

## Abbreviations

The following abbreviations are used in this manuscript:

Gen-AI	Generative Artificial Intelligence
GAI	Generative Artificial Intelligence
PGAIU	Problematic Generative Artificial Intelligence Use
UGAIU	Unethical Generative Artificial Intelligence Use
CFI	Comparative Fit Index
GFI	Goodness-of-Fit Index
RMSEA	Root Mean Square Error of Approximation
SRMR	Standardized Root Mean Square Residual
